# Regulatory T Cells Play a Role in Determining the Tumourigenicity of the Intestinal Stem Cell Niche

**DOI:** 10.1016/j.gastha.2024.09.014

**Published:** 2024-09-26

**Authors:** Ana Padilha, Emma Jones, Scott Cutting, Andrew Godkin, Awen Gallimore, Lee Parry

**Affiliations:** 1 European Cancer Stem Cell Research Institute, School of Biosciences, Cardiff University, Cardiff, UK; 2 Systems Immunity University Research Institute/Division of Infection and Immunity, School of Medicine, Cardiff, UK

Intestinal stem cells (ISCs) are key players in maintaining the function of epithelium and barrier protection. It is now apparent that endogenous and exogenous influences impact on ISC number, plasticity, and behavior to maintain intestinal and mucosal homeostasis. Immune cells play a crucial role in this process by shaping how the ISC niche responds to such infleunces.^1^ As adaptive cluster of differentiation (CD) 4 positive (CD4^+^) T cells producing the cytokines IFNγ or IL-13 restrain ISC proliferation whilst driving differentiation.^2^ Conversely, IL-10-producing Foxp3^+^ regulatory T cells (Treg) maintain ISC renewal in the stem cell niche by controlling CD4^+^T cell activation.^2^ A better understanding of this immune: ISC/epithelia crosstalk is crucial to developing translational approaches to disease and healthy aging. As anti-inflammatory foxp3^+^ regulatory T cell (Treg) cells maintain the *Lgr5+* ISCs, the cell of origin for many colorectal cancers (CRCs).^3^ Tregs may limit CRC risk by maintaining ISC homeostasis or controlling inflammation or, conversely, promote disease progression by inhibiting anti-tumour specific T cells (reviewed in^4^). A greater understanding on the impact of immune perturbations to the ISC population may shed light on gut health and how CRC risk may be altered. To better understand this relationship, we combined mouse models to examine the impact of Treg on ISC numbers, tumour initiation and the T cell response.

To explore at which stage Tregs begin to exert their influence we used the *Apc*^*+/min*^ mouse model where, akin to CRC, tumourigenesis is driven by spontaneous *Apc* loss driving progression from aberrant crypt foci (ACF) to adenomas. At 60 days, we observed a significant increase in the density of Tregs within microadenomas (*Apc*^*+/min*^T) compared to surrounding normal tissue (*Apc*^*+/min*^N)(Figure 1A and Figure A1A and B), supporting what has previously been demonstrated in *Apc*^*+/min*^ adenomas.^5^ Next to examine the tumour initiation phase, we used the conditional *Lgr5CreER*^*T2*^*Apc*^*flx/flx*^ model to examine Treg cells alongside conditional ISC *Apc* deletion (*Apc*^*ΔISC*^) (Figure A1C). Fifteen days following *Apc*^*ΔISC*^ a significant increase in Tregs was observed around ACF in small intestine and large intestine (LI) compared to control mice (Figure 1A–C & Figure A2A) and overall *FoxP3* expression (Figure 1D). A significant increase in the proportion and number of Treg cells was observed in the small intestine intraepithelial layer and lamina propria layers, LI intraepithelial layer, spleen, and mesenteric lymph nodes indicating local and systemic Treg expansion following *Apc*^*ΔISC*^ loss (Figure 1E–G and Figure A3A–C). This data raised the possibility that Tregs are actively involved during the tumour establishment period.

**Figure 1:**
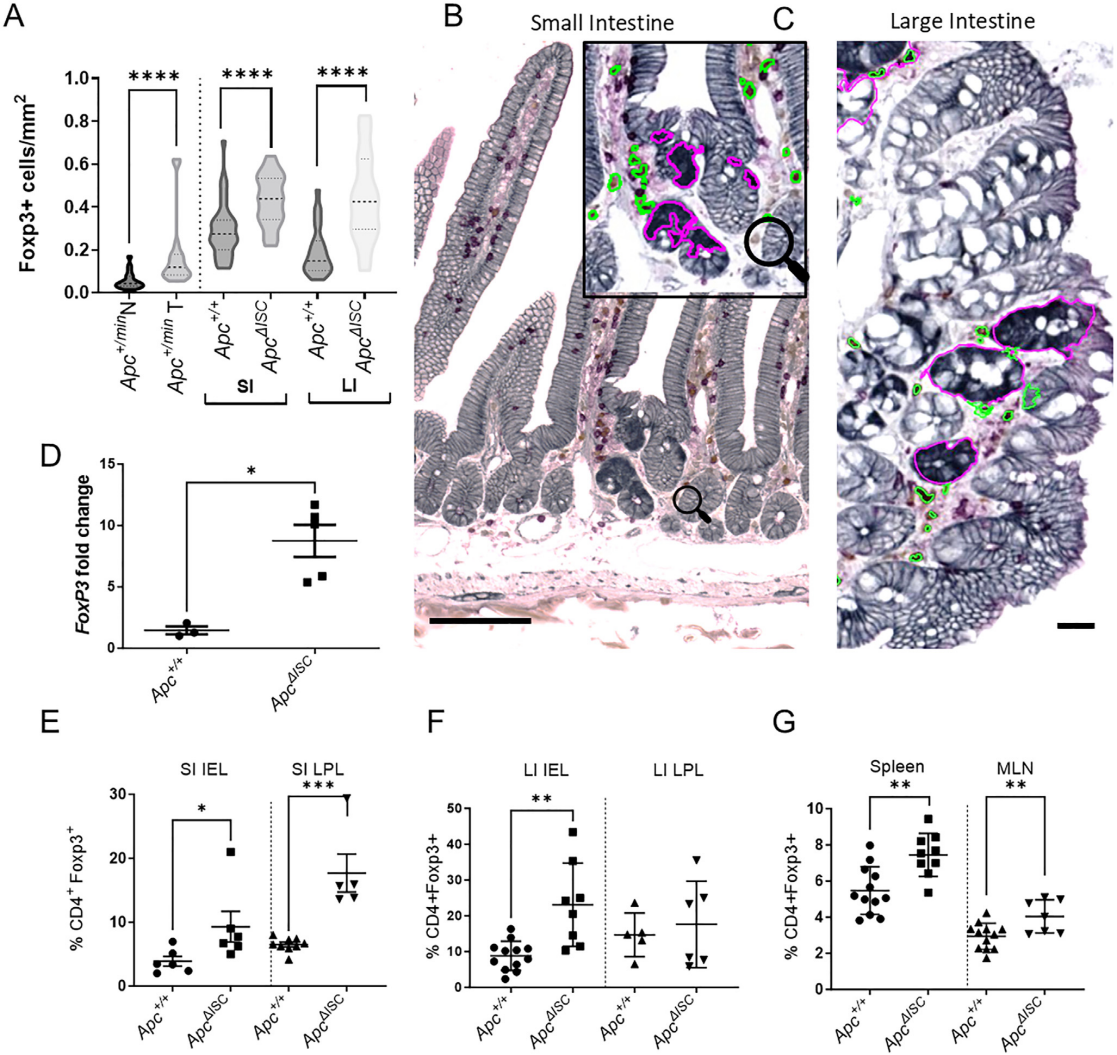
Tregs accumulate around intestinal tumours within 15 days of *Apc* ISC loss. (A) Density of Treg cells in area surrounding intestinal tumour tissue (as identified by nuclear β-catenin staining; N > 4 animals). (B) small and (C) large intestine IHC image showing Treg cells (Green - CD3+FoxP3+) surrounding unrecombined WT crypts or Wnt driven *Apc*^*ΔISC*^ ACF (purple – nuclear β-catenin+) 15 days following *Apc* deletion (scale bar 20μm). (D) Increased relative gene expression of *FoxP3* in SI. (E–G) Flow cytometry quantification demonstrating compartment specific *Apc*^*ΔISC*^ changes of CD4+ and CD4+Foxp3+ populations (Mann-Whitney U test; *P* ∗<0.05, ∗∗<0.01, ∗∗∗<0.001 or ∗∗∗∗<0.0001). ACF, aberrant crypt foci; CD, cluster of differentiation; ISCs, intestinal stem cells; SI, small intestine; Treg, foxp3^+^ regulatory T cell.

To test this, the *Lgr5creER*^*T2*^*Apc*^*flx/flx*^ mice were crossed with *FoxP3*^*DTR*^^6^ mice to examine Treg cell depletion, following diphtheria toxin injection (Treg^DEP^), alongside *Apc*^*ΔISC*^ (Figure A1C). At day 15, *Apc*^*ΔISC*^Treg^DEP^ mice had on average fewer ACF in the intestine that was significant in the LI (Figure 2A), supporting a role for Tregs in promoting ISC tumourgenicity. A more detailed immune cell analysis demonstrated that Treg^DEP^ resulted in a significant increase in CD4^+^ and CD8^+^ T cells; however, this was not augmented in *Apc*^*ΔISC*^Treg^DEP^ mice (Figure 2B–C). Further in-depth immune analysis indicated that the significant elevation of CD4^+^granzymeB^+^, CD4^+^IFNγ^+^ and CD8^+^IFNγ^+^ observed in Treg^DEP^ mice was significantly reduced in the *Apc*^*ΔISC*^ Treg^DEP^ setting (Figure A4A–C, other nonsignificant data not shown). To determine whether Treg depletion unleashes an anti-tumour immune response to *Apc*^*ΔISC*^, through induction of IFNγ-producing, Granzyme B + T cells capable of killing ISCs^7^^,^^8^ we administered CD4 or CD8 depleting antibodies. Surprisingly, quantification of ACF established that both CD4^+^ and CD8^+^ depletion significantly reduced ACF number in *Apc*^*ΔISC*^ mice and failed to rescue this phenotype in the *Apc*^*ΔISC*^Treg^DEP^ mice (Figure 2D). This decrease in ACF due to CD4+ cell neutralisation is in keeping with an ISC homeostasis role for the CD4+ Treg cells.^2^ While the impact of CD8 neutralisation may reflect the loss of CD8+αEβ7+ T-cells, as αEβ7 allows them to bind E-cadherin (to remain within the intestinal epithelium), down regulate Wnt activity and direct ISC fate via integrin signalling.^9^ Thus, it appears that CD8^+^ and possibly Foxp3^-^CD4^+^ T cells may also promote ISC tumourigenicity. The data does, however, indicate that any pro-tumorigenic effect of Tregs following *Apc* deletion in the ISC is unlikely to be due to their role in suppressing anti-tumour CD4^+^ or CD8^+^ T-cells.

**Figure 2:**
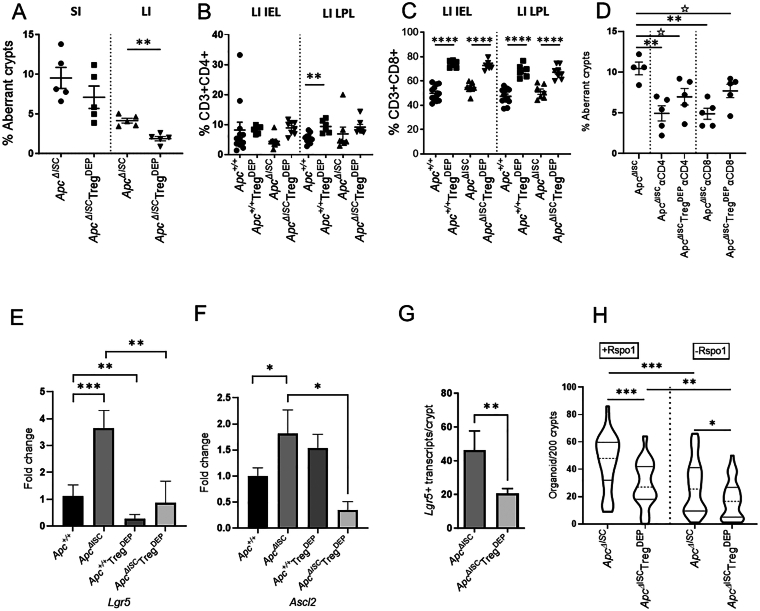
Reduction in intestinal tumourigenesis due to Treg depletion is due to altered immune:ISC crosstalk rather than immune elimination. (A) Quantification of ACF 15 days following ISC *Apc* loss and/or Treg depletion. (B and C) Cell quantification demonstrating *Apc*^*ΔISC*^*Treg*^*DEP*^ specific immune changes in LI IEL (left) & LI LPL (right). (D) Quantification of ACF following neutralization of CD4+ or CD8+ cells. (E–G) Downregulation of ISC markers in *Lgr5* & *Ascl2* due to Treg depletion. (H) Quantification of organoids derived *ex vivo* from *Apc*^*ΔISC*^Treg^DEP^ intestinal crypts at day 5 indicating loss of functional ISCs. (Mann-Whitney U test; *P*: 1-tailed ^☆^<0.05; 2-tailed ∗<0.05, ∗∗<0.01, ∗∗∗<0.001 or ∗∗∗∗<0.0001). IEL, intraepithelial layer; ACF, aberrant crypt foci; CD, cluster of differentiation; ISCs, intestinal stem cells; LI, large intestine; LPL, lamina propria; Treg, foxp3^+^ regulatory T cell.

We next considered that the impact of Treg^DEP^ on the emergence of ACF may reflect their role in maintaining the normal ISC pool, as Treg depletion began 3 days prior to *Apc*^*ΔISC*^ (Figure A1C). Expression analysis in mice at day 5 indicated a significant decrease in expression of the ISC genes *Lgr5 and Ascl2* in the *Apc*^*ΔISC*^Treg^DEP^ mice and number compared to controls (Figure 2E–G and Figure A2B). To confirm a reduction in functional ISC numbers we performed *ex vivo* organoid culture using crypts from *Apc*^*ΔISC*^Treg^DEP^ mice utilising R-spondin (Rspo1; essential for *Apc*^*+/+*^ organoids) to distinguish between WT and mutant ISCs. Quantification demonstrated a significant reduction in total (+Rspo1 – *Apc*^*+/+*^ *& Apc*^*ΔISC*^) and *Apc*^*ΔISC*^ (-Rspo1) organoids because of Treg depletion (Figure 2H). This ISC reduction due to Treg loss is in keeping with previously published data.^2^ With our new data demonstrating that Treg support of ISCs increases the number of cells capable of initiating a tumour following an *Apc* mutation. Evidence that CD8+ and Foxp3^-^CD4+ T cells also influence tumorigenesis suggests that the pro-tumourigenic effects of Tregs may extend to their indirect effects on the behaviour of these T cells.

Collectively these data highlight a previously unappreciated association between Treg and altered CRC risk. Intestinal cellular homeostasis is maintained by the ISCs, which can either expand in presence of Tregs or diminish in their absence. This cross-talk may facilitate a rapid response to inflammation or injury to ensure epithelial integrity. These findings demonstrate that the role of Tregs in maintaining tissue homeostasis and responding to environmental changes is a major influence on the risk of cancer development. New therapeutic approaches that aim to reduce Treg cells to promote anti-tumour immune CRC responses^10^ should also consider the impact on the CRC stem cells and normal ISC population.

## Figures and Tables

**Figure A1:**
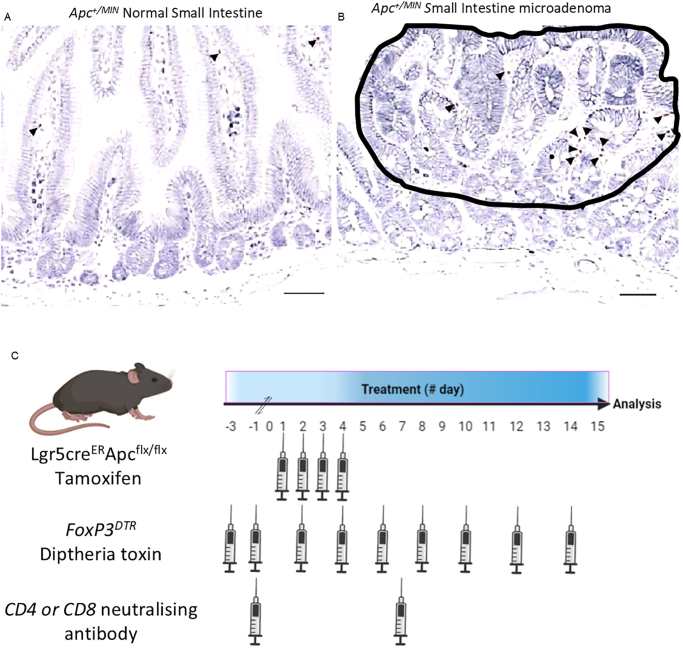
IHC image of (A) normal and (B) microadenoma (black outline; β-catenin-grey) tissue from a 60-day old *Apc*^+/min^ mouse showing adenoma specific accumulation of Treg cells (black arrows FoxP3+-brown; scale bars = 20). (C) Schematic indicating agents and administration regimes for conditional transgenes and neutralising antibodies.

**Figure A2:**
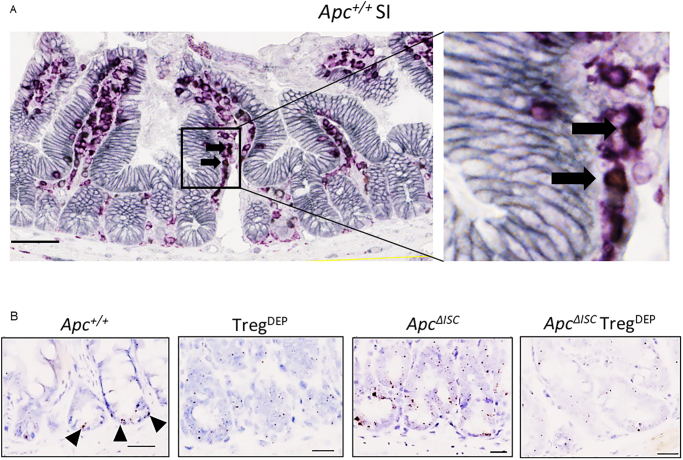
(A) IHC image of normal intestine demonstrating low residual numbers of Treg cells in the absence of *Apc* loss (Black arrows indicate Treg cells; β-catenin-grey, CD3-purple & FoxP3-brown; scale bar = 100μm). (B) RNAscope® images indicating gain of *Lgr5* transcripts upon *Apc*^*ΔISC*^ opposed to their loss following Treg depletion (*Lgr5* transcripts highlighted by black arrows in left panel, scale bar = 50μm).

**Figure A3:**
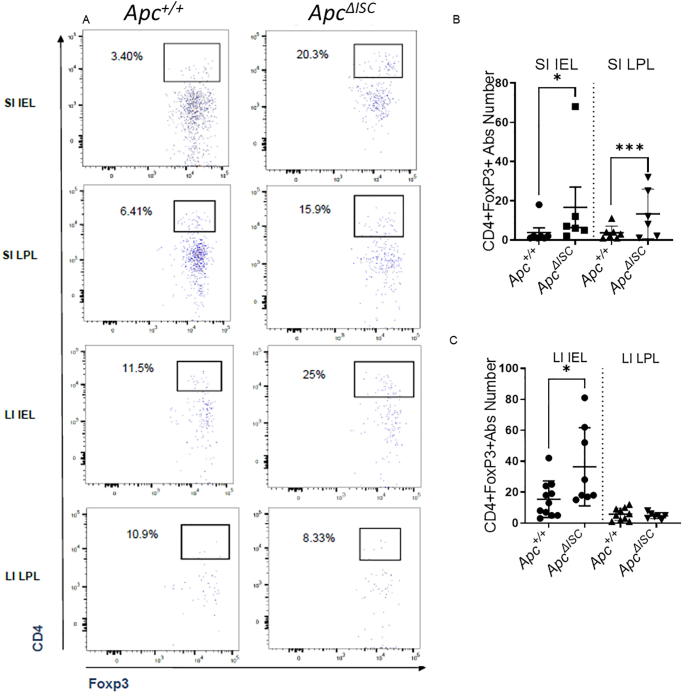
(A) Flow cytometry plots demonstrating number of Tregs in intestine (CD4+FoxP3+ cells)(*Apc*^*+/+*^ - left hand column & *Apc*^*ΔISC*^ right hand column). (B and C) Quantification of absolute numbers by flow cytometry of CD4+FoxP3+ in intestine 15 days following ISC *Apc* loss (*P* ∗<.05).

**Figure A4:**
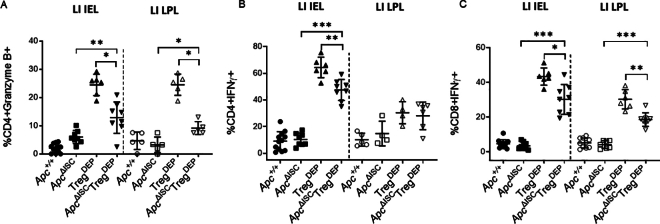
(A–C) Significant changes observed upon flow cytometry of large intestine IEL (left panels) and LPL (right panels) immune cells 15 days following manipulation of ISC *Apc* and Treg status (*P* ∗<.05 & ∗∗<.01).

## **Supplementary materials**

FinalSupplementalMaterial
