# Supplementary material for: Regulatory T Cells Play a Role in Determining the Tumourigenicity of the Intestinal Stem Cell Niche
Source: Gastro Hep Adv. 2024 Sep 26;4(2):100559. doi: 10.1016/j.gastha.2024.09.014 (PMC11773477; doi:10.1016/j.gastha.2024.09.014)
Supplement: FinalSupplementalMaterial [file mmc1.docx]

**SUPPLEMENTAL INFORMATION**

**Regulatory T Cells play a role in determining the tumourigenicity of the Intestinal Stem Cell Niche**

Ana Padilha^1^, Emma Jones^2^, Scott Cutting, Andrew Godkin^2^, Awen Gallimore^2^*, Lee Parry^1^*

Animal Models

*Lgr5Cre*^ERT2^ *Apc*^fl/fl^ *Foxp3*^DTR^ mice were bred in a specific pathogen-free (SPF) barrier facility and housed in conventional facilities during procedures. Equivalent numbers of male and female mice between 10 and 15 weeks old were entered at the start of experiments.

Induction of the *Lgr5Cre*^ERT2^ transgene was achieved by administering a single intraperitoneal injection (IP) of 10mg/ml tamoxifen (TAM; 80 mg/kg; Sigma-Aldrich) mixed in corn oil (Sigma-Aldrich) for four consecutive days. For *in vivo* depletion of Foxp3+ Treg cells 15 μg/Kg diphtheria toxin (DT; Sigma-Aldrich), diluted in sterile phosphate buffered saline (PBS; ThermoFisher Scientific) was IP injected every other day for 15 days, starting a day prior to TAM injection. Depleting CD4-specific antibodies were produced in house (1). Mice were injected with 2 mg of anti-CD4 antibodies (clones YTS-191 and YTA-3) prior to TAM and again one week later. CD8+ cell depletion was achieved by IP injection of 250ug of the depleting anti-CD8 monoclonal antibody (clone YTS-169) (Bioxcell), a day prior to TAM injection and 100 μg one week later. All experiments were repeated at least twice.

*Intestinal Epithelial Cells Extraction for Gene Expression Analysis*

Following dissection, a section of the small intestine (between the 5th and 12^th^ cm) was opened longitudinally and cut into small fragments. Fragments were washed gently with HBSS containing HEPES (1:100) before the supernatant was discarded and 10 ml 8 mM EDTA (Sigma-Aldrich) was added for incubation on ice for 30 min. Following incubation, tubes containing the fragments were shaken vigorously and the supernatant collected into a fresh 50ml tube containing 10 ml complete RPMI 1649 medium supplemented with GlutaMAX (Life Technologies), penicillin/streptomycin (Thermo Fisher Scientific) and 10% Foetal Bovine Serum (FBS) (Thermo Fisher Scientific). This process was repeated before fragments were filtered through a 70 μm cell strainer (MACS) and supernatant collected and spun down at 1000 rpm for 5 minutes. The resulting pellets were re-suspended in 1ml RNA later (Sigma-Aldrich). RT-qPCR was performed as previously described (2). Oligonucleotide sequences and probes used for relative quantification are available upon request.

*Immunohistochemistry (IHC) and cellular analysis*

Small and large intestines were collected, opened longitudinally, rolled and fixed in ice-cold 10% formalin (Sigma-Aldrich) for 24 hours before processing into wax blocks. Sections were cut at 5 μm thickness and rehydrated. Staining was performed using the anti-rat (mouse adsorbed), anti-mouse, and anti-rabbit ImmPRESS HPR kits (Vector Labs) according to the manufacturer’s instructions. The following antibodies were used for immunohistochemistry: anti-β-catenin (BD Biosciences), anti-Foxp3 (eBioscience), anti-CD4 (eBioscience), anti-CD8 (eBioscience) and anti-CD3 (DAKO). Samples were incubated with secondary antibody and signal visualized with an enzyme-specific, chromogenic colour, according to manufacturer’s recommendations. Tissue was counterstained in hematoxylin, dehydrated and mounted in distyrene, plasticizer, xylene mountant (DPX; Sigma-Aldrich). To quantify histological sections the images that were acquired in the Zeiss Axio Scan.Z1 slide scanner were analysed using the ZEN image analysis software. At least four mice per cohort were analysed. To analyse the total tumour burden, the whole crypts touching the basement membrane with positive nuclear B-catenin staining (aberrant crypts), from both small and large intestines, were counted. The paraffin embedded tissues were cut into three serial sections separated by 120 μm distance to avoid the quantification of the same aberrant crypts. A total of five mice were analysed. Data was normalised by counting the total number of whole crypts touching the intestinal basement membrane. Partial tumour burden was analysed by assessing the number of whole crypts with positive nuclear B-catenin in 100 crypts of the first 5 cm of the small intestine. The average number of aberrant crypts was calculated and the mean across the cohorts was determined. A minimum of four mice were analysed.

The number of Treg was determined by co-B-catenin-CD3-Foxp3 IHC staining. For *Apc^+/min^* tumours CD3+FoxP3+Tregs were counted within the adenoma as identified by B-catenin staining. For the *Lgr5creApc^flx/flx^* mice Tregs were quantified around unrecombined *Apc^flx/flx^* or *Apc^∆ISC^* crypts as identified by β-catenin nuclear staining in the: (1) small intestine - the region between the crypt:villus junction and the basement membrane and (2) large intestine-region immediately surrounding the normal or aberrant crypts with a minimum number of five fields of view of at least 50μm^2^. Cells were quantified from 25 normal and 25 aberrant whole crypts. A minimum of four mice were analysed. The average number of positive cells was calculated and the mean across the cohorts was determined.

*Isolation of Lymphocytes and Flow Cytometry Analysis*

Spleen and MLN were homogenized using the back of a 1ml syringe plunger through a 70 µm cell strainer (MACS). Red blood cells in spleen were lysed using RBC lysis buffer (Sigma) Small (SI) and large (LI) intestines were open longitudinally, cut into small fragments and placed into 50 ml tubes with HBSS containing HEPES buffer. Intra epithelial (IEL) and lamina propria (LPL) lymphocytes were obtained as previously described (3).

Cells were washed in PBS, stained using an aqua amine-reactive viability dye (LIVE/DEAD Aqua, Invitrogen), then washed. Fc receptors were blocked with anti-CD16/32 (clone 93; eBioscience) before staining with surface antibodies to CD3 (AF488, eBioscience), CD4 (BV421, BD Bioscience) and CD8 (APC-H7, BD Bioscience). For intracellular staining, cells were stimulated in 96-well plates with 50 ng/ml phorbol myristate acetate (PMA; Sigma – Aldrich) and incubated with 1 µl/ml ionomycin (Sigma – Aldrich) and 5 µg/ml brefeldin A (Sigma – Aldrich) for 3h. Prior to intracellular staining with with Foxp3 (PeCy7, eBioscience), granzyme B (APC, Biolegend), IL-17A (PE, BD Pharmingen) and IFNγ (PE-Pharmingen) specific antibodies, cells were fixed/permeabilized according to the manufacturer's protocol (Foxp3-staining kit; eBiosciences). Data were acquired on a NovoCyte ® 3000 cytometer and analysed using FlowJo version 10 and NovoExpress software.

*RNAscope*

*In situ* detection of *Lgr5* mRNA transcripts using the RNAscope kit 2.5 HD Assay-BROWN (Advanced Cell Diagnostics, Hayward, CA, USA) was performed according to the manufacturer's protocol. Positive staining was determined by brown punctate dots and their quantification was performed at the crypt base in a total of 30 crypts per mouse; the average number of brown dots was calculated and the mean across the cohorts was determined.

*Ex vivo* analysis

Mice were harvested and 200 intestinal crypts per well were plated in Matrigel in Intesticult™ organoid culture medium (Stemcell technologies, UK). Medium was replaced every 2 days and organoid number determined at day 7 (Gelcount, Oxford Optronix, UK).

*Statistical analysis*

Data were analysed using Graph Pad Prism 10 software as indicated in figure legends. All relevant comparisons where significant differences exist are indicated on graphs (2-tailed Mann-Whitney ** P<.05, ** P<.01, *** P<.001 & **** P<.0001*).

Acknowledgements

Authors would like to thank Mark Bishop, Jolene Twomey and Carys Holmes for technical support during this project.

For the purpose of Open Access, the author has applied a CC BY public copyright license to

any Author Accepted Manuscript (AAM) version arising from this submission.

Supplementary References

1. Golgher-D. et al. EJI. 2002,
2. May S. et at. J Pathol. 2018, **245**: 270-281.
3. Uhlig H. et al. J Immunol. 2006, **177(9)**: 5852-5860.
